# Effectiveness of Fenbendazole and Metronidazole Against *Giardia* Infection in Dogs Monitored for 50-Days in Home-Conditions

**DOI:** 10.3389/fvets.2021.626424

**Published:** 2021-03-26

**Authors:** Lavinia Ciuca, Paola Pepe, Antonio Bosco, Simone Mario Caccio, Maria Paola Maurelli, Anna Rosa Sannella, Alice Vismarra, Giuseppe Cringoli, Laura Kramer, Laura Rinaldi, Marco Genchi

**Affiliations:** ^1^Department of Veterinary Medicine and Animal Production, University of Naples Federico II, Naples, Italy; ^2^European Union Reference Laboratory for Parasites, Department of Infectious Diseases, Istituto Superiore di Sanità, Rome, Italy; ^3^Department of Veterinary Science, University of Parma, Parma, Italy

**Keywords:** *Giardia*, dogs, 50 days post-treatment, fenbendazole, metronidazole, assemblage

## Abstract

A field trial performed in-home conditions was conducted on 24 dogs naturally infected with *Giardia*, in order to compare the efficacy of fenbendazole and metronidazole. Animals were allocated in groups randomly in order to obtain two groups of 12 dogs each with similar parasitic loads of *Giardia* cysts: dogs in Group A were treated with fenbendazole (Panacur®, Intervet Italia Srl) administered at the dose of 50 mg/kg orally once a day for 5 consecutive days, dogs in Group B were treated with metronidazole (Flagyl®, Zambon Italia Srl) administered orally at the dose of 50 mg/kg, once a day for 5 consecutive days. All the dogs that were shedding *Giardia* cysts after the first treatment (Day 0) were retreated (either at Day 7 or at Day 14 or at Day 21) until a negative result was obtained with the same treatment. Additionally, all the dogs were re-examined at Day 50. All the dogs were tested for the presence of *Giardia* cysts using a fecal flotation method (FLOTAC). The percent efficacy of the treatments (A and B) was calculated at each sampling point (Days 7, 14, 21, and 50) as reduction in mean *Giardia* cysts. After the first therapy, on day 7, 4/12 (33.3%) dogs tested positive for *Giardia* cysts in the Group A and 5/12 (41.7%) in the Group B. Efficacies at (Days 7, 14, 21, and 50) of the treatments against *Giardia* infection were 80.9, 94, 100, and 97% in the Group A and 70.8, 99, 100, and 97.1% in the Group B. Statistically significant differences were not observed between the efficacy of Fenbendazole and Metronidazole against infection by *G. duodenalis* (*P* = 0.686). Molecular analysis revealed full homology (i.e., 100% with JN416550) with the canine specific assemblage D in six positive dogs. Different hypotheses might explain the re-appearance of the *Giardia* cysts in some dogs after treatment, e.g., re-infection from the home environment, the correct medication given by the owners, the diet, as well as treatment failure, but also biological issues related to the intermittent excretion of *Giardia* cysts.

## Introduction

*Giardia duodenalis* (syn. *Giardia lamblia, Giardia intestinalis*) is the causative agent of giardiosis and one of the most commonly found parasite in dogs throughout the world (1, 2). Molecular studies have shown that *G. duodenalis* comprises at least eight distinct genetic assemblages (A–H), of which assemblages C and D are found exclusively in dogs, while *Giardia* assemblages A and B have zoonotic potential (3–5).

Infection by *G. duodenalis* in dogs has a worldwide distribution with prevalence rates that vary according to the test population, the diagnostic method used and the geographical area (6, 7). In Italy, prevalence values range between 11.1 and 28.9% in northern (8–10), between 6.4 and 21.4% in central (11–14) and between 7.7 and 14.2% in southern regions (15, 16).

The diagnosis of *Giardia* infection in dogs may pose a challenge, due to a low infectious dose and marked persistence of cysts in the environment as well as a fluctuating excretion of the cysts in the feces that makes the monitoring of this parasite problematic (17, 18). Traditional tools for identification of *Giardia* cysts include fecal smear, zinc sulfate flotation technique (17, 19), immunofluorescence assay (IFA; the gold standard), immunochromatography, enzyme-linked immunosorbent assays (ELISA), and molecular analyses that permit the different *G. duodenalis* assemblages to be distinguished (17, 20, 21). Among the copromicroscopic techniques, a recent study reported a high sensitivity and specificity of FLOTAC technique with zinc sulfate for diagnosis of *Giardia* cysts infection in dogs (19, 22).

Also the control of *Giardia* infection in dogs is prone to a number of issues. Several compounds have been tested against *Giardia* infections in dogs as benzimidazoles, in particular fenbendazole, and metronidazole that poses activity against the parasite (23–28). Other studies reported the efficacy of ronidazole, nitazoxanide, azithromycin, tinidazole, and ipronidazole and other drugs such as quinacrine, furazolidone, in reducing *Giardia* cysts shed by infected dogs (17, 29–32). Another option recommended by the European Scientific Counsel Companion Animal Parasites (ESCAAP) is the combination of febantel (a prodrug metabolized *in vivo* to fenbendazole)/pyrantel/praziquantel at the standard deworming dose (15.0 mg/kg of febantel, 14.4 mg/kg pyrantel, 5.0 mg/kg praziquantel) repeated once daily for 3 days (33). Recently, secnidazole, a molecule that is used for the treatment of giardiosis in humans, has been reported as an effective drug for the treatment of clinical canine giardiosis (34). However, fenbendazole and metronidazole are used routinely to treat giardiosis in dogs and are the only compounds registered in most European countries.

Resistance to antiparasitic drugs has been often suggested to explain treatment failure due to the incomplete parasite removal following treatment or other underlying diseases (e.g., inflammatory bowel diseases, bacterial overgrowth, coinfection with other organisms). Shampooing of dogs (e.g., with a product containing chlorhexidine digluconate) at the beginning and at the end of antiprotozoal treatment is also recommended to reduce re-infections through fecal material on the fur (30). Despite treatment recommendation with fenbendazole for eliminating *Giardia* cysts in dogs, currently, unpublished data from veterinary practices are showing a low efficacy of this drug in eliminating the infection. Taking into account that the ESCCAP Guidelines recommends fenbendazole (1x/d, 50 mg/kg for 3 days) and metronidazole (2x/d, 25 mg/kg for 5-7 days) for the treatment of *Giardia* infections in dogs (33), the purpose of the present study was to reassess the efficacy of these two specific drugs for the treatment of giardiosis in owned dogs, monitored for 50 days in home-conditions.

## Materials and Methods

### Animal Testing and Study Design

A field trial performed in-home conditions was conducted in 2018–2019 on 24 owned dogs naturally infected by *Giardia* spp. and living in the Campania region, southern Italy. The dogs included in the study were referred to the Laboratory of Parasitology and Parasitic Diseases at the Department of Veterinary Medicine and Animal Production, University of Naples Federico II, for coproparasitological analysis. Dog owners were informed about the study protocol and they gave their consent to inclusion of their pets. Animals were allocated in groups randomly in order to obtain two groups of 12 animals each with similar parasitic loads of *Giardia* cysts: group A-fenbendazole (mean cyst per gram of feces-CPG = 20,995) and group B-metronidazole (CPG = 18,580). The cysts were enumerated using a fecal flotation method (Flotation, Translation and Centrifugation) FLOTAC technique (see Laboratory Analysis). In addition, the dogs had the following characteristics: 11 males (five long-haired and six short-haired dogs) and 13 females (six long-haired and seven short-haired dogs), aged between 2 months and 5 years, living indoor but with access to outdoors. All the dogs included in the study did not live in the same household with other animals. Exclusion criteria were animals treated with any antiparasitic drug in the 2 weeks before, animals with any aggressive behavior and animals showing clinical signs of any other diseases. For each animal included in the study, data regarding age, fur length, clinical signs and consistency of fecal samples were recorded and analyzed. For ethical reasons, no untreated control-group of animals was available. Dogs in Group A were treated with fenbendazole tablets (Panacur®, Intervet Italia Srl) administered at the dose of 50 mg/kg orally once a day for 5 consecutive days. Dogs in Group B were treated with metronidazole tablets (Flagyl®, Zambon Italia Srl) administered orally at the dose of 25 mg/kg, orally, twice a day for 5 consecutive days. All the dogs started the first treatment at Day 0. All the dogs that were shedding *Giardia* cysts after the first treatment were retreated (either at Day 7 or at Day 14 or at Day 21) until a negative result was obtained with the same treatment. Additionally, all the dogs were re-examined at Day 50. Treatment efficacy was evaluated based on the mean CPG found in the fecal samples on Day−5 (pre-treatment) and on Days 7, 14, 21, and 50 (post-treatment). Additionally, each dog was subjected to shampooing with chlorhexidine digluconate at the beginning and at the end of each treatment applied. Furthermore, each owner was suggested to follow some basic rules to avoid the reinfection by cleaning and drying the environment, the use of clean utensils for feed and water and proper disposal of feces (33).

### Laboratory Analysis

Dogs were initially identified and verified as infected with *Giardia* spp. and then subsequently analyzed at Day−5 (pre-treatment) and at Days 7, 14, 21, and 50 after the first treatment applied (Day 0). The fecal samples analyzed at each sampling point were represented by pools collected on a daily basis for 2 days (prior to the treatment date). Analyses were performed within 24 h of sampling. At each sampling day the feces for all dogs were tested for the presence of *Giardia* cysts using the FLOTAC technique with an analytic sensitivity of 1 cyst per gram (CPG) of feces (19). Each sample was analyzed using zinc sulfate (specific gravity = 1.350).

Fifteen samples collected at Day−5 were fixed in 2.5% potassium bichromate (dilution 1:4) and processed for molecular studies. Briefly, genomic DNA was extracted using the FastDNA SPIN Kit for feces (MP Biomedicals, Solon, Ohio, USA). A nested PCR assay was used to amplify a 511 bp fragment of the beta-giardin gene according to a published protocol (35). DNA from axenic cultures of *Giardia duodenalis* strains of assemblage A (WB, genotype AI) and assemblage B (GS, genotype BIV) was used as positive controls in PCR assays. PCR products were purified using spin columns (QiaQuick PCR Purification kit –Qiagen) and sequenced from both strands.

### Treatment Efficacy

The percent efficacy of the treatments (A and B) was calculated at each sampling point from the trial (Days 7, 14, 21, and 50) according to the following formula [adapted from (36)].

$$\begin{array}{lll} \text{\%Efficacy} & = & \frac{\text{Mean CPG day}-5-\text{Mean CPG day }7}{\text{Mean CPG day }-5}\times \;100\\ \text{CPG} & = & \text{cysts per gram of feces}. \end{array}$$

### Statistical Analysis

Statistical analysis was performed using Windows SPSS® (version 17.0). The non-parametric Mann-Whitney U test was used to determine the level of significant difference between groups of treatment (A, B).

### Assessment Criteria and Clinical Outcome

An arbitrary scoring system (from 1 to 4) was used and a score assigned to each dog, based on fecal consistency and clinical symptoms (lethargic attitude, growth retardation, weight loss, vomiting and flatulence) as follows: (1) formed feces and no clinical symptoms, (2) formed feces and clinical symptoms, (3) soft stools and clinical symptoms, and (4) diarrhea and clinical symptoms.

## Results

Table 1 shows dog's data (gender, age, fur length, clinical score), *Giardia* cysts per gram (CPG) of feces (mean, standard deviation) and efficacy (%) of treatment with fenbendazole (Group A) and metronidazole (Group B) at the different study Days (Day−5, Day 7, Day 14, Day 21, and Day 50).

**Table 1:** Groups A-Fenbendazole and B-Metronidazole: dog's data (gender, age, fur length, clinical score), *Giardia* cysts per gram (CPG) of feces (mean, standard deviation), and efficacy (%) of the treatment at the different study days.

**Dog ID**	**Gender (Male/Female)**	**Age (months/years)**	**Fur length (Long/Short)**	**Clinical score**	***Giardia*** **cysts per gram (CPG) of feces**				
					**Day-5 (Pre-T)**	**Day 7 (T1)**	**Day 14 (T2)**	**Day 21 (T3)**	**Day 50**
**GROUP A (FENBENDAZOLE)**									
F1	M	2 mos	L	4	22,300	0	0	8,360	0
F2	F	4 yrs	S	3	15,600	0	0	0	0
F3	M	8 mos	L	1	19,200	21,600	0	0	0
F4	M	2 yrs	S	1	7,830	1,230	0	0	0
F5	F	1 yr	S	3	18,894	0	0	0	0
F6	F	3 mos	L	4	24,600	16,828	0	0	0
F7	F	1 yr	L	2	21,080	0	0	0	0
F8	M	9 mos	S	3	31,984	0	0	0	0
F9	F	2 yrs	S	2	22,824	0	0	0	12,576
F10	M	3 yrs	L	1	16,854	0	0	0	0
F11	F	7 mos	S	3	48,000	0	0	0	0
F12	M	3 yrs	L	1	2,780	8,400	0	0	0
				**Mean CPG**	20,995.5	4,004.8	0.0	696.7	1,048.0
				**SD**	11390.1	7564.0	0.0	2413.3	3630.4
				**%Efficacy**	**NA**	**80.9%**	**100%**	**97.0%**	**95.0%**
**GROUP B (METRONIDAZOLE)**									
M1	F	4 yrs	S	4	19,654	0	0	0	0
M2	F	4 yrs	S	3	11,900	0	0	0	0
M3	F	8 mos	L	1	10,900	0	0	0	0
M4	M	2 yrs	S	1	21,234	0	0	0	0
M5	F	2 yrs	L	2	10,848	0	0	0	0
M6	M	9 mos	S	1	21,280	0	0	0	6,546
M7	M	3 yrs	S	1	18,732	560	156	0	0
M8	F	1 yr	L	2	15,832	17,560	0	0	0
M9	M	6 mos	S	3	17,459	18,981	0	0	0
M10	F	5 yrs	L	2	17,178	17,562	0	0	0
M11	M	5 mos	L	4	32,178	10,400	0	0	0
M12	F	7 mos	S	2	25,765	0	1,987	0	0
				**Mean CPG**	18,580.0	5,421.9	178.6	0.0	545.5
				**SD**	6224.6	8161.8	571.3	0.0	1889.7
				**%Efficacy**	**NA**	**70.8%**	**99.0%**	**100%**	**97.1%**

*Pre-T (before treatment); T1 (first treatment); T2 (second treatment) T3 (third treatment). Clinical score: (1) formed feces and no clinical symptoms, (2) formed feces and clinical symptoms, (3) soft stools and clinical symptoms, and (4) diarrhea and clinical symptoms*.

Parasitological results at Day−5 showed mean values of 20,995 *Giardia* CPG (minimum 2,780 CPG, maximum 48,000 CPG) in the Group A (fenbendazole) and 18,580 *Giardia* CPG (minimum 10,848 CPG, maximum 32,178 CPG) in the Group B (metronidazole). On Day 7, cysts of *Giardia* spp. were found in 33.3% (4/12; 95% Confidence Interval (CI) = 11.3–64.5) of the dogs in the Group A and in 41.7% (5/12; 95% CI = 16.5–71.4) of the dogs in the Group B.

The efficacy of fenbendazole was 80.9% at Day 7, 100% at day 14, 97.0% at day 21, and 95.0% at day 50. The efficacy of metronizazole was 70.8% at Day 7, 99.0% at Day 14, 100% at Day 21, and 97.1% at Day 50. Overall, the efficacies of Fenbendazole and Metronidazole against the infection by *G. duodenalis* were not significantly different (*P* = 0.686).

No other co-infections with intestinal parasites were found at copromicroscopic examinations. Briefly, 6 dogs from Group A and 5 dogs from Group B remained negative until Day 50 after they received the first treatment at Day 0. Eight dogs (four from each group), received the second treatment at Day 7 (after the first therapy at Day 0), and two other dogs (one from each group) were retreated for the second time at Day 21 from the Group A and Day 14 from the Group B. Moreover, in the Group B, there was one dog that received three treatments during the study at Days 0, 7 and 14. Finally, two dogs (one from each group), that had received the first treatment at Day 0, were still shedding *Giardia* cysts at the last study Day (Day 50).

### Assessment Criteria and Clinical Outcome

At Day−5, feces were unformed to diarrhoeic in dogs belonging to both treatment groups but no adverse responses due to the medications were observed. 33.3% of the dogs had score 1, 25% score 2, 25% score 3, and 16.7% score 4. Regarding the dogs that tested positive for *Giardia* after the treatment (13/24), five belonged to the first category (38.6%), four to the second category (30.8%), one to the third category (7.7%), and three to the fourth (23%).

### Molecular Characterization

*Giardia* DNA could be amplified only from six of the 15 fecal samples tested, three from the Group A (dog's ID numbers = F1, F6, F8) and three from the Group B (dog's ID numbers = M4, M7, M11). The beta-giardin amplification products were sequenced and compared, using BLAST, with all available sequences in the GenBank database. This revealed full homology (e.g., 100% with JN416550) with the canine specific assemblage D in the six positive dogs. The lack of amplification in the remaining samples is likely due to cyst damage resulting from storage conditions.

## Discussion

During the last 60 years, a number of chemotherapeutic agents have been introduced and are still in use in the therapy for giardiosis (37). Unfortunately, most drugs used have considerable adverse effects such as vomiting, bloody diarrhea, abortion, neurological dysfunctions and so they are contraindicated (38). In this context, studies on chemotherapeutic agents play a fundamental role in the rationale for the treatment of giardiosis.

Several benzimidazoles (39, 40), and in particular fenbendazole (23), or the combinations of febantel/fenbendazole with other compounds proved to be effective (24, 25, 41). Metronidazole, a nitroimidazole, and fenbendazole are used routinely to treat giardiosis in dogs (30). Ronidazole showed a good antiprotozoal effect against *Giardia* in dogs (30) and oxfendazole showed a significant decrease in the number of cysts (40).

The findings of the present study did not reveal a significant difference of efficacy between the drugs used (fenbendazole 80.9% vs. metronidazole 70.8% at Day 7). Moreover, after applying the first treatment, both drugs failed to eliminate the *Giardia* cysts in all the dogs. Re-appearance of the cysts in the feces could be attributed to a re-infection, treatment failure, the correct medication given by the owners, the diet, as well as biological issues related to the intermittent excretion of *Giardia* cysts. However, there are no specific information of the dog's environment at home if any attempt was made by the owner to clean up contaminated areas. Faure et al. (42), reported a significant difference of efficacy between fenbendazole and metronidazole (30.3% vs. 91.9%). In our study, fenbendazole showed lower efficacy than that reported by Zajac et al. (26), who obtained 90% efficacy (26). However, it is important to note that in our present study both drugs showed 100% efficacy after two consecutive cycles of treatments, i.e., at Days 14 in the Group A and at Day 21 in the Group B.

Fifteen (62.5%) out of 24 dogs treated from both groups (eight dogs from Group A and seven dogs from Group B) were tested negative for *Giardia* cysts after the first therapy at Day 7. Moreover, there were dogs from both groups A and B, thus showing a persistent infection that has been cleared either at Day 14 or at Day 21 after two or three treatments of 5 days each. It could be possible, that some *Giardia* remained at low abundance in the intestine of these dogs that were still positive after the first treatment applied, or for the dogs that needed to be treated three times until a negative result was obtained, or perhaps we were facing with a treatment failure due to drug resistance. However, future investigations are needed, in order to establish the availability of the drugs in the blood and the capacity of these to eliminate the *Giardia* cysts by *in vitro* methods.

In addition, two dogs from each group (A and B) remained negative after the first therapy (Day 0), until Day 50, when turned positive. In these cases, it was suggested that reinfections from the environment are the most common cause for treatment failure (25, 30) considering that the prepatent period can be as short as 4 days (33). Also, the presence of cysts on the animal's fur, associated with the resistance of the parasite to disinfectants (chlorhexidine gluconate in our study) and environmental conditions and with stress, may explain reinfections (30). Since this is a field trial performed in-home conditions we were not sure about the failure of cleaning and bathing due to the lack of compliance by the owner, thus, the use of hygiene measures such as chlorhexidine digluconate shampoo before and after the treatment might have a considerable influence on our results representing a major issue of this study. Moron-Soto et al. also reported that dogs re-shed cysts following a brief period after antiparasitic treatment when no disinfection or cleaning of their enclosures was performed (31).

Some experimental studies have evaluated also the efficacy of the oral administration of probiotics together with albendazole (38), therefore during the treatment the veterinarian can recommend the administration of probiotics that play a synergistic role with the drug regenerating the intestinal flora. However, in the present study, none of the dogs received a special diet, or probiotics supplements during and after the therapy. Moreover, there are few recent studies that showed that the microbial diversity was not altered by fenbendazole administration, which is in contrast to metronidazole which significantly altered microbial structure and diversity (43–47). Administration of metronidazole to healthy dogs caused significant changes in the microbiome, some of which persist following discontinuation of the drug with unknown clinical effects. Furthermore, some dogs on metronidazole can get neurotoxicity (48, 49). However, in the present study, no adverse responses due to the medications were observed including transient neurological signs due to metronidazole.

There have been two reports examining the effects of secnidazole on *Giardia* in dogs. One, reported 100% efficacy of cyst reduction in two groups of six hospitalized dogs (50). The other one, showed that at 43 days after the first treatment, all 9 dogs were considered to have normal stools, but 4 of the 9 puppies were positive for *Giardia* antigen (34). Bearing these facts in mind, perhaps secnidazole applied twice, 2–3 weeks apart, could be an alternative and easier treatment, with a better compliance as well for treating *Giardia* infection. Further data are needed to prove its efficacy both in field and experimental studies.

Finally, genotyping of *Giardia* demonstrated the host-specific assemblage D in all positive dogs, in agreement with other studies in Italy (12, 35). However, our study presented limitations due to the sample size and due to the poor and inappropriate storage conditions to ensure the effective isolation of DNA from fecal samples. Therefore, further research on distinguish *G. duodenalis* strains, are needed, in order to perform a realistic estimation of zoonotic risk (51).

Based on the findings, as expected in a trial with a small number of individuals in-home conditions, and especially for a parasite with the characteristics of *Giardia* (i.e., having a direct infection and short prepatent period), the authors could only speculate that treatment alone is not sufficient for controlling *Giardia* infection.

A main limitation of our study is based on the small sample size (no. = 12 dogs), the absence of a control group and the allocation of the animals in the two treatment Groups based only on individual *Giardia* CPG assessed at Day−5. However, the limited number of dogs in our study was mainly due to type of study (field trial in-home conditions) that requires a full compliance of both vet practitioners and dog owners. Ethical issues were the main reasons for not including a control group in such a kind of field trial performed in-home conditions. In a similar study, Mirò et al. (52) assessed the efficacy of fenbendazole and mebendazole using 10 per group. As a consequence, in the allocation phase we could not consider other key epidemiological parameters as age, fur length and style of living. Different hypotheses might explain the re-appearance of the *Giardia* cysts in some dogs after treatment, e.g., re-infection from the home environment, the correct medication given by the owners, the diet, as well as treatment failure, but also biological issues related to the intermittent excretion of *Giardia* cysts. Therefore, due to the uncontrolled parameters in this field trial performed in-home conditions, future studies are warranted to produce conclusive evidence for the evaluation of integrated approaches needed for the treatment of dogs naturally infected by *Giardia*.

## Data Availability Statement

The raw data supporting the conclusions of this article will be made available by the authors, without undue reservation.

## Ethics Statement

The animals used in the present study were sampled following approval by the animal ethics and welfare committee of the University of Naples Federico II (in Italian, Comitato Etico-scientifico per la Sperimentazione Animale dell' Università di Napoli Federico II; protocol number PG/20170055343). Written informed consent was obtained from the owners for the participation of their animals in this study.

## Author Contributions

MG, LR, LC, LK, SMC, ARS, and GC contributed to the conception and design of the study. PP, AB, AV, and MPM organized the database and performed the statistical analysis. MG, LR, and LC wrote the manuscript. All authors contributed to manuscript revision, read, and approved the submitted version.

## Conflict of Interest

The authors declare that the research was conducted in the absence of any commercial or financial relationships that could be construed as a potential conflict of interest.
